# Experimental biofilm models for pharmacokinetic and pharmacodynamic investigations: bridging *in vitro*, *ex vivo* and *in vivo* systems

**DOI:** 10.1093/jac/dkag091

**Published:** 2026-03-06

**Authors:** Stephanie Supparitsch, Markus Zeitlinger

**Affiliations:** Department of Clinical Pharmacology, Medical University of Vienna, Vienna 1090, Austria; Department of Clinical Pharmacology, Medical University of Vienna, Vienna 1090, Austria

## Abstract

Biofilm-associated infections represent a major therapeutic challenge due to reduced antimicrobial susceptibility and the limited predictive value of conventional pharmacokinetic/pharmacodynamic (PK/PD) indices with clinical outcome. A wide spectrum of experimental models has been developed to study biofilms, ranging from simple *in vitro* assays to *ex vivo* tissue-derived systems and *in vivo* infection models. Each category provides distinct advantages: *in vitro* platforms enable high-throughput compound screening and measurement of biofilm-specific indices such as MBIC and MBEC; *ex vivo* models preserve host tissue architecture and allow investigation of topical therapies and therapeutic windows; and *in vivo* systems are indispensable for analysing host–pathogen interactions and systemic PK/PD relationships. No single model is sufficient to replicate clinical biofilm complexity, but combined use and progressive standardization can improve translational value. This review provides a structured overview of available models, their PK/PD readouts and their strengths and limitations, aiming to guide model selection in preclinical biofilm research and antimicrobial development.

## Introduction

Biofilms are highly structured communities of microorganisms that adhere to surfaces and are embedded in a self-produced extracellular polymeric substance (EPS) matrix.^1,2^ The EPS matrix is central to biofilm functionality, providing structural integrity, hydration and a barrier against environmental stressors. It consists of polysaccharides, proteins, lipids and nucleic acids, providing mechanical stability and protection to the biofilm. The matrix is separated by open water channels, which have the purpose of supplying nutrients and removing metabolic waste products.^1–3^ It also facilitates horizontal gene transfer allowing for a higher occurrence in biofilms which consequently enables the exchange of genetic material, including antibiotic resistance genes.^2^ The definition of biofilms extends beyond simple surface attachment, as these microbial communities exhibit cooperative behaviours, complex communication and metabolic interdependence, distinguishing them from planktonic (free-floating) microorganisms.^2,3^

Several authors have proposed definitions to describe the defining characteristics of biofilms. More recent conceptual frameworks emphasize that biofilms are not restricted to surface-associated growth but can also exist as non-surface-attached aggregates, a phenotype frequently observed *in vivo* in both clinical and environmental settings. Accordingly, bacterial aggregates embedded in a self-produced extracellular matrix are now commonly regarded as biofilms irrespective of their attachment to biotic or abiotic surfaces.^4–6^

### Development of biofilms

**Figure 1.**
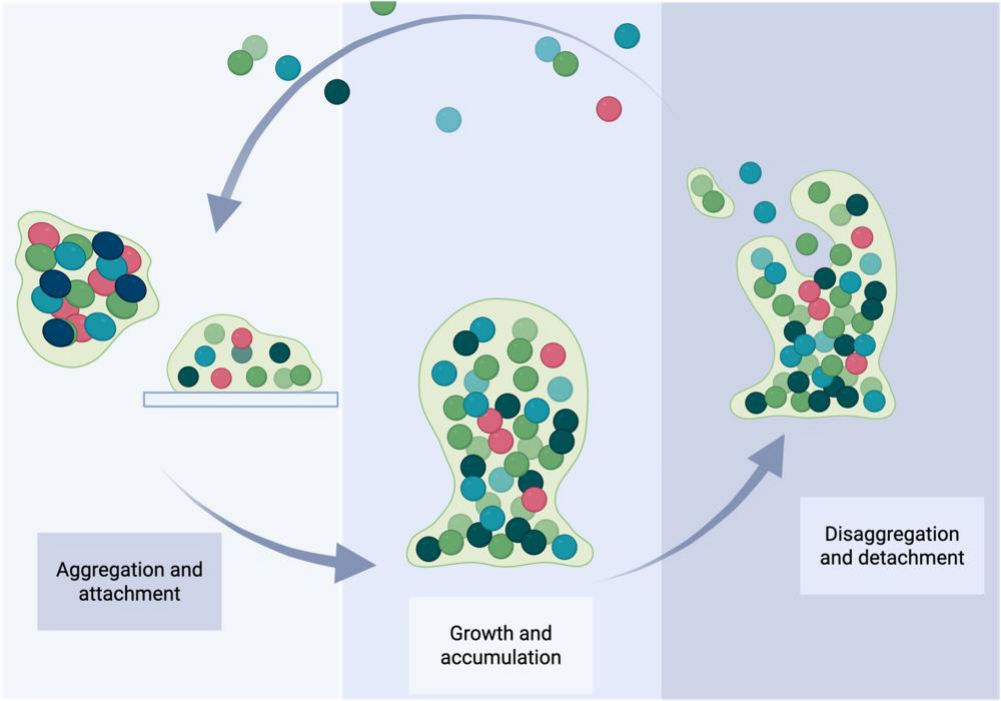
Stages of biofilm development. Schematic representation of biofilm development according to an inclusive three-event model. Aggregation and attachment describe the initial association of microbial cells through cell–cell aggregation and/or attachment to biotic or abiotic surfaces, accompanied by early extracellular polymeric substance (EPS) production. During growth and accumulation, biofilm biomass expands through cell division and recruitment, leading to structured communities embedded in an EPS matrix and characterized by spatial heterogeneity. Disaggregation and detachment encompass the release of single cells or multicellular aggregates from the biofilm, enabling the dissemination and colonization of new sites. These events are not strictly sequential and may overlap depending on environmental conditions and system context. Created in https://BioRender.com.

Biofilm development is commonly described as a three-stage lifecycle: (i) attachment/formation, (ii) maturation and (iii) dispersal, depicted in Figure 1. For practical and mechanistic purposes, the attachment/formation stage is often subdivided into an initial reversible attachment followed by irreversible attachment once EPS production stabilizes the community. During maturation, biofilms develop three-dimensional structures and pronounced microenvironments, shaped by gradients in nutrients, oxygen and waste products. Dispersal can be triggered by environmental stress, nutrient limitation or quorum-sensing-regulated programs, enabling dissemination and colonization of new sites.^4–6^

### Relevance of biofilms

Biofilms are relevant across health, industry and the environment; however, this review focuses on infection- and device-associated biofilms where antimicrobial pharmacology is central. In medicine, biofilms contribute to chronic and relapsing infections (e.g. chronic rhinosinusitis, chronic wound infections, otitis media and dental plaque) and are frequently implicated in device-associated infections such as catheters, implants and prostheses.^1,2,7,8^ In the intensive care setting, central venous catheters (CVCs) represent a major source of biofilm-associated infections, as biofilm formation on intraluminal and extraluminal surfaces can occur within 24 h after insertion and is detected in the majority of long-term indwelling catheters.^9,10^ In a retrospective study after three consecutive years of 466 cerebrospinal fluid (CSF) shunt operations in neonates, Pople *et al*.^11^ noted 46 cases in which a biofilm infection was detected, i.e. 10%.

Biofilm infections are also highly relevant in chronic airway disease; for example, people with cystic fibrosis (CF) can develop persistent biofilm-associated airway infections (notably with *Pseudomonas aeruginosa*) within mucus-rich environments.^12^

Biofilm-associated infections are aggravated by the fact that the protective nature of the EPS matrix allows the microorganisms to exhibit increased resistance to antibiotics and the host immune system, posing significant challenges not only to the process of diagnosing the infection but also to successive antimicrobial treatment.^1,2,7,13^

### Challenges in biofilm research and experimental modelling

Investigating biofilms presents several challenges due to their complexity and dynamic nature. One primary challenge is the understanding of the biofilm structure and characteristics themselves. Replicating biofilm conditions *in vitro* is difficult, as laboratory models often fail to fully capture the heterogeneity and environmental influences present in natural or clinical settings, such as the absence of host immune responses or reduced oxygen availability.^14^ This limitation can be overcome by studying biofilms *in vivo*.^2^ The spatial and metabolic diversity within biofilms further complicates analysis, as traditional microbiological methods are often insufficient to study the localized interactions and gradients.^15^ However, the development and use of new technologies such as matrix-assisted laser desorption/ionization (MALDI) imaging or single-cell RNA sequencing (scRNA-seq) is promising in terms of bringing new insights into the underlying heterogeneity and gene expression patterns.^15^ Advanced imaging techniques, such as confocal laser scanning microscopy (CLSM) and high-resolution electron microscopy, have been developed to study biofilm structure and dynamics.^1^ CLSM enables the observation and analysis of living organisms, as they do not have to be dehydrated or dried.^13,16^ Since biofilms consist mainly of water, this method allows for more physiologically relevant imaging and has reshaped our foundational understanding of biofilm architecture.^7,13^

### Antimicrobial treatment challenges in biofilm infections

Clinically, the main challenge is the antimicrobial treatment of biofilm-associated infections. The pharmacokinetic (PK) and pharmacodynamic (PD) behaviour of antibiotics changes significantly when targeting these biofilm-associated infections as several factors might contribute to the reduced efficacy of antibiotics in biofilms. Limited antibiotic penetration and delayed transport occur within biofilms not because of restricted diffusion through water but due to interactions of antibiotics with EPS components (e.g. binding to polysaccharides or extracellular DNA), enzymatic inactivation within the matrix and reaction-diffusion processes that reduce antibiotic availability in deeper biofilm layers.^17–19^ Additionally, biofilm bacteria exhibit reduced metabolic activity, rendering them less susceptible to antibiotics that target actively growing cells, such as beta-lactams or ciprofloxacin.^7,18,20^ Moreover, persister cells, a subpopulation within the biofilm, can tolerate high antibiotic concentrations without undergoing genetic resistance, leading to chronic and relapsing infections.^7,20^ Recent studies have demonstrated that combination therapy, including beta-lactams with aminoglycosides or fluoroquinolones with macrolides, can enhance antibiotic efficacy against biofilms by targeting multiple bacterial survival mechanisms simultaneously.^20^

### Pharmacodynamic endpoints used in biofilm research

Conventional susceptibility endpoints such as the MIC and MBC often fail to capture the reduced susceptibility of bacteria in biofilms. Therefore, biofilm-specific pharmacodynamic (PD) endpoints have been introduced. The minimum biofilm inhibitory concentration (MBIC) denotes the lowest antibiotic concentration that prevents further biofilm growth or biomass accumulation, typically quantified by optical density, metabolic activity or imaging-based readouts. The minimum biofilm eradication concentration (MBEC) is an operational endpoint describing the antibiotic concentration associated with eradication of a preformed biofilm under defined experimental conditions. Importantly, ‘eradication’ can be defined as the absence of detectable regrowth or as achieving a specified log reduction (e.g. ≥3-log decrease) and is influenced by the assay’s limit of quantification, plated volume and recovery method (e.g. sonication, scraping or homogenization).^21,22^

These endpoints are PD measures. PK/PD relationships emerge when biofilm PD endpoints (e.g. MBIC/MBEC, time–kill kinetics and regrowth suppression) are linked to antibiotic exposure metrics such as AUC, C_max_ or time above a threshold concentration within relevant compartments (e.g. sputum, tissue and device lumen).^23–25^

#### Linking models to PK/PD inference: what needs to be represented

PK/PD inference in biofilms depends not only on the PD endpoint (e.g. MBIC/MBEC, time–kill kinetics and regrowth suppression) but also on whether the experimental system captures exposure features that determine drug action at the biofilm site. Key determinants include (i) time-varying exposure rather than fixed concentrations, (ii) spatial gradients in nutrients/oxygen and antimicrobial exposure that create heterogeneous microenvironments, (iii) compartmentalization (e.g. mucus, tissue and device lumen) that defines the relevant site of action and (iv) sampling strategy and readout alignment, since biomass, cfu and regrowth endpoints reflect different pharmacodynamic objectives. Models therefore differ primarily in how well they represent exposure dynamics and the site of action, which in turn determines whether they support qualitative ranking, quantitative exposure–response modelling or translation to clinically relevant targets.^7,19,21,25–27^

In summary, biofilms represent a critical area of research due to their ubiquity and diverse implications. Their structural complexity, adaptive capabilities and resistance mechanisms pose significant challenges for researchers, necessitating innovative approaches to better understand and manage biofilms in various contexts.

The current review aims to (i) summarize biofilm model systems across *in vitro*, *ex vivo* and *in vivo* settings and (ii) critically evaluate how model-specific exposure characteristics, compartments and readouts enable or constrain PK/PD inference for antibiotics in biofilms.

Accordingly, PK/PD analysis in this review is structured hierarchically: *in vitro* models are discussed in terms of their ability to isolate exposure–response relationships, *ex vivo* models in terms of local pharmacodynamic validation and *in vivo* models in terms of integrated, translational PK/PD assessment.

## Introduction to the model classification section

**Figure 2.**
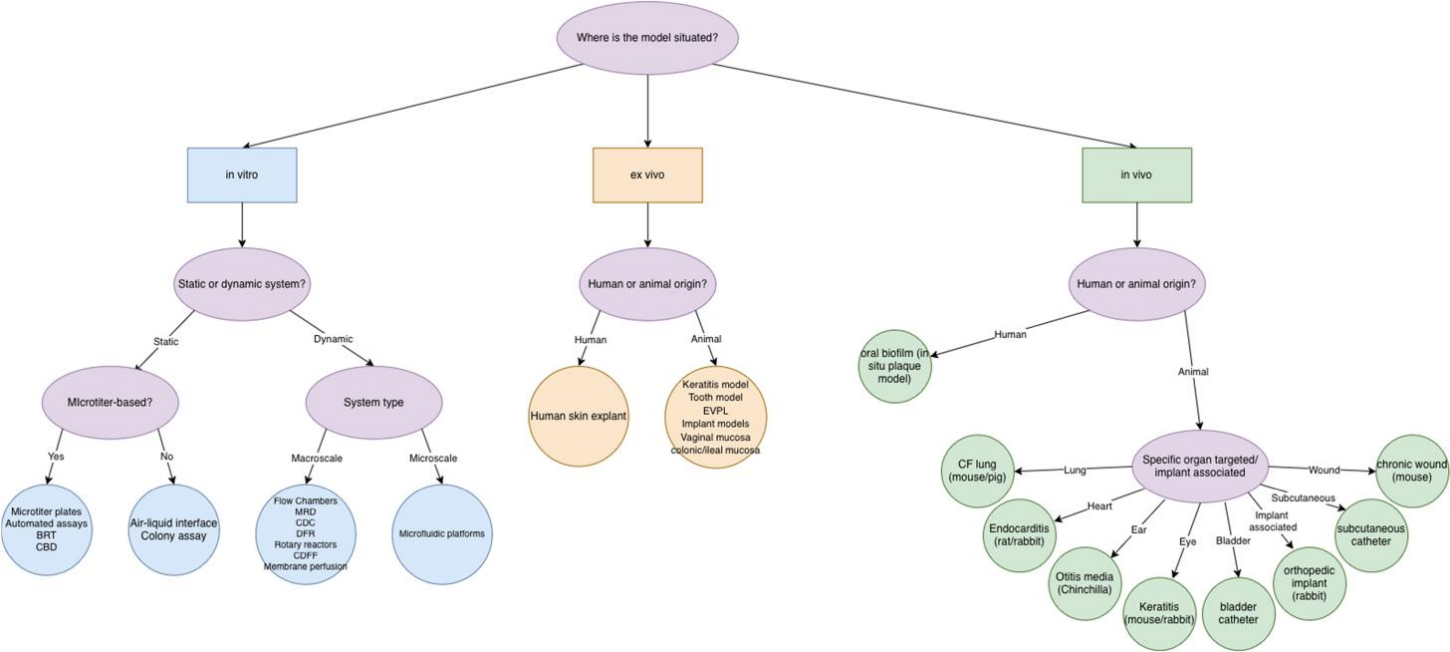
Graphic overview of biofilm models.

To facilitate the selection of suitable biofilm models for pharmacokinetic and pharmacodynamic (PK/PD) studies, a technically oriented decision flowchart (Figure 2) has been developed. This chart categorizes commonly used biofilm models based on key criteria such as complexity, degree of automation, tissue origin (*in vitro*, *ex vivo*, *in vivo*) and standardization.

The following section is structured according to this framework and presents biofilm models in line with their technical characteristics, experimental applicability and relevance for PK/PD assessments. Already established as well as emerging models are considered, covering a broad spectrum of anatomical sites and material-associated contexts.

## In vitro *models*

*In vitro* models constitute a fundamental pillar of biofilm research by enabling reproducible and mechanistically controlled investigations of microbial behaviour, antimicrobial efficacy and resistance development. Based on the degree of automation and fluid dynamics, these models can be categorized into the following groups.

### Static systems

#### Microtitre plate-based model systems

##### Microtitre plates/automated assays/Biofilm Ring Test (BRT)/Calgary Biofilm Device

Microtitre plate (MTP)-based systems form the cornerstone of high-throughput *in vitro* biofilm research. All widely used static assays, including the Calgary Biofilm Device (CBD) and the Biofilm Ring Test (BRT), are direct adaptations or extensions of the traditional MTP format, reinforcing the centrality of microtitre platforms in biofilm methodology. In these systems, biofilms grow either directly on the well walls and bottoms of 96-well plates or on material discs/coupons placed inside multi-well plates of various formats (e.g. 6-, 12-, 24- or 96-well).^28–33^ These batch reactor-like setups lack flow, resulting in a dynamic decrease in nutrient availability and metabolite accumulation over time, unless fluids are regularly refreshed.^34^

The benefits of MTP-based models, namely, low cost, minimal reagent use and the capacity for parallel testing, have led to their widespread application in screening antimicrobial compounds, studying biofilm-deficient mutants and assessing antibiofilm material coatings.^34–37^ Notably, these systems can be tailored by modifying surface materials or environmental parameters (e.g. oxygen, CO_2_ and humidity), enhancing their flexibility.^34^

Both CBD and BRT, while differing in detection methods, are built entirely on microtitre plates. The CBD employs a lid with pegs immersed in wells, allowing for standardized biofilm cultivation and treatment workflows. It remains a gold standard for minimal biofilm eradication concentration (MBEC) determinations.^38–40^ The BRT utilizes magnetic beads within the culture medium and tracks their immobilization to quantify early biofilm formation. The MTP format supports this method’s automation and reproducibility.^41–44^

Microtitre plates also serve as versatile scaffolds for more complex adaptations. They can accommodate medically relevant surfaces such as silicone or titanium discs, which are analysed by techniques like scanning electron microscopy (SEM) after cultivation.^45–47^ This adaptability allows modelling of mixed-species or inter-kingdom biofilms, such as *Candida albicans* with *Staphylococcus aureus* or *S. epidermidis*, mimicking clinically challenging infections.^48–51^

Nevertheless, MTP-based assays share common limitations. As they generally operate as closed, static setups, these environments fail to accurately reflect the dynamic and heterogeneous nature of *in vivo* biofilms. Additionally, common biomass stains like crystal violet cannot distinguish between viable and dead cells or between biofilm and sedimented planktonic cells. Advanced quantification methods such as MTT or viable plate counting can partially address these challenges.^52–54^

In summary, many widely used static screening assays rely on microtitre plate-based architectures because they enable parallelization, standardization and automation. However, important complementary approaches exist outside the microtitre format [e.g. colony biofilms, air–liquid interface (ALI) systems and flow-based reactors] and can capture biological features not represented in closed batch setups.

#### Non-microtitre plate-based model systems

##### Colony biofilm assay/air–liquid interface assay

The colony biofilm assay involves depositing bacterial suspensions onto membrane filters on agar plates. Bacteria form structured colonies with gradients of nutrients, oxygen and waste products, closely mimicking nutrient-limited biofilm microenvironments. Nevertheless, the absence of shear forces and continuous nutrient supply limits its physiological relevance and translational value.^14,19,55^

Air–liquid interface (ALI) biofilm models simulate host–pathogen interactions at mucosal surfaces by cultivating bacteria on semipermeable membranes exposed to air on one side and nutrient media on the other, often overlying epithelial cell layers. This structure supports stratified biofilm development under low-shear, nutrient-gradient conditions similar to the respiratory or sinus mucosa. In one such model, *P. aeruginosa* was cultured on collagen-coated membranes atop airway epithelial monolayers to study chronic rhinosinusitis, showing robust and reproducible biofilm formation at the host interface.^56^ ALI systems have also been used to assess antimicrobial pharmacodynamics: aerosolized tobramycin was applied to mature *P. aeruginosa* biofilms on bronchial epithelial cells, allowing dynamic analysis of bacterial burden, cytokine release and epithelial barrier function over time, demonstrating the model’s potential for PK/PD investigations.^57^ In a further application, longitudinal proteomic analysis of ALI-grown *P. aeruginosa* biofilms revealed time-dependent shifts in virulence and nutrient metabolism, confirming the model’s utility for mechanistic and temporal biofilm studies.^58^

Static models are most informative for determining biofilm-specific PD endpoints under standardized conditions (e.g. MBIC/MBEC, time–kill or regrowth suppression at fixed concentrations). Their main limitation for PK/PD inference is that exposure is typically approximated as constant and bulk, whereas clinically relevant exposure is dynamic and compartment-dependent; consequently, static systems are best suited for comparative ranking and mechanistic screening and require additional design elements (e.g. repeated medium exchange or staged dosing) when used for more quantitative exposure–response analysis.^34,53^

### Dynamic systems

#### Macroscale dynamic systems

##### Flow chambers/modified Robbins device/Center for Disease Control and Prevention biofilm reactor/drip flow reactor/rotating wall vessel/constant depth film fermenter

Dynamic biofilm models, by contrast, simulate physiologically relevant environments through continuous flow and nutrient exchange. These open systems fall into two main reactor categories: continuous flow stirred tank reactors (CFSTRs) and plug flow reactors (PFRs).^34^ CFSTRs ensure uniform environmental conditions, while PFRs replicate spatial gradients, mimicking physiological heterogeneity.^34^

Among the most established systems is the modified Robbins device (MRD), composed of a flow channel fitted with removable plugs hosting biofilm substrates. MRDs have been extensively used to test surface-modified materials, simulate laryngeal prostheses and study antibiotic lock therapy.^59–61^ MRD systems allow variable flow rates and shear conditions, enabling the assessment of antimicrobial efficacy under clinically relevant hydrodynamics.^61,62^

The CDC biofilm reactor and the drip flow reactor (DFR) are also widely used and recognized by ASTM standards (E2196-07, E2647-08). The CDC reactor supports high-shear conditions, ideal for testing disinfectants and antimicrobial efficacy, while the DFR enables low-shear environments with slow fluid movement, ideal for studying spatial heterogeneity and nutrient gradients in biofilms.^18,63–67^

Rotary reactors, including the rotating disc and annular biofilm reactors, introduce controlled shear forces independently of flow rate. This allows fine-tuned experimentation on mechanical stress responses in biofilms of *P. aeruginosa*, *S. aureus* and multispecies communities. They are particularly useful for high-throughput disinfection or cleaning efficacy testing.^68–70^

The constant depth film fermenter (CDFF), often used in dental biofilm research, maintains biofilms at a fixed thickness via mechanical scraping, simulating the tongue’s cleaning motion. This system has been employed to evaluate both antimicrobial coatings and surface modifications.^71,72^

More novel systems include a membrane-based dynamic model developed by Duckworth *et al.*^73^, which permits polymicrobial biofilm culture on semipermeable membranes under perfusion from below. This design allowed for complex community dynamics to emerge, mirroring clinical wound infections and enabled antimicrobial efficacy testing with higher physiological fidelity compared to static models.

While dynamic models enhance environmental control, simulate real-life conditions and facilitate temporal analysis, they are technically demanding, costly and often low throughput. Moreover, the absence of chamber isolation in many systems limits the ability to test numerous variables simultaneously. Nevertheless, their capacity to reproduce *in vivo*-like gradients in nutrients, oxygen and metabolic by-products, together with spatial and temporal gradients in antimicrobial exposure and clinically relevant mechanical forces (e.g. shear stress), makes them critical tools in translational biofilm and PK/PD research.

#### Micro-scale dynamic systems

##### Microfluidic biofilm platforms

Microfluidic biofilm platforms have emerged as powerful tools to replicate physiologically relevant conditions with single-cell resolution. These systems are composed of micro-scale channels (typically 50–500 µm wide and 30–250 µm deep), enabling precise control over flow rates (0.1–50 µL/min), nutrient gradients, shear stress and chemical cues under highly laminar flow conditions.^74,75^ Photolithographic fabrication allows fine-tuning of architecture and reproducibility across experiments, although device manufacturing can be complex and costly.^53,76^

The compact dimensions of microfluidic chambers support real-time visualization via optical microscopy, making them ideal for monitoring biofilm dynamics, dispersion and treatment effects at the single-cell level.^44,53^ These systems are compatible with both off-chip and on-chip analytical techniques and are especially suited for studying biofilm response to antimicrobial gradients or signalling molecules.^44,53^

In one example, Lee *et al.*^75^ used a microfluidic chip to control dispersin B delivery to preformed biofilms, observing successful dispersal when combined with antibiotics. Wright *et al.*^77^ applied a wound-like nutrient gradient to mimic infection environments and observed chemotactic responses driving biofilm maturation.

Notably, Jung *et al*.^78^ developed a shear-free microfluidic chamber that supports non-surface-attached biofilms embedded in a matrix, simulating mucosal or intracellular biofilms. Furthermore, integrated platforms like the BioFlux system (Fluxion Biosciences) incorporate microfluidic channels into MTPs for parallel testing of up to 96 biofilms, facilitating high-throughput antimicrobial screening.^53,79,80^

Additionally, co-culture models combining epithelial cells with bacteria (e.g. *Escherichia coli* biofilms on HeLa monolayers) have been developed to better reflect host–microbe interactions.^81,82^ While technically demanding, microfluidic platforms offer exceptional flexibility and insight into the spatial, temporal and molecular aspects of biofilm development, making them valuable tools for experimental microbiology and drug discovery.

*In vitro* biofilm models provide reproducible, scalable and cost-effective systems for studying microbial behaviour and antimicrobial efficacy under controlled conditions. Despite their reductionist nature and limited physiological relevance, they remain indispensable for mechanistic and PK/PD investigations, particularly through standardized formats such as the Calgary Biofilm Device, microfluidic platforms and flow systems that allow precise analysis of antibiotic penetration, tolerance and eradication dynamics.^14,34,53,73^

Dynamic flow systems and microfluidic platforms better support PK/PD-oriented questions by enabling defined delivery and washout, control of shear and, in some configurations, spatial antimicrobial gradients, thereby approximating key exposure features relevant for biofilm response. These systems are particularly useful for analysing time-dependent killing, penetration-delayed effects, regrowth niches and the impact of microenvironmental gradients on local susceptibility. Their limitations are primarily practical: lower throughput, higher technical complexity and often non-trivial sampling strategies, which can restrict dense dose-time designs needed for robust quantitative exposure–response modelling.^14,53,73^

### Overview

Models are grouped according to their operational principle (static, dynamic or cell-based) and degree of automation as summarized in Table 1. Each system allows assessment of specific pharmacodynamic endpoints such as minimal biofilm inhibitory concentration (MBIC), minimal biofilm eradication concentration (MBEC) or biofilm-associated viability.

**Table 1.** Overview of commonly used *in vitro* biofilm model systems for PK/PD investigations

Model	Category	Host/species	Pathogen (s)^a^	PK/PD aspect	PK/PD parameters	References
Microtitre plate (MTP)	Static/automated	None	*P. aeruginosa*, *S. aureus*, *Burkholderia cepacia*, staphylococci	Standard screening	MIC, MBIC, MBEC	^28–37^
Calgary Biofilm Device (CBD)	Static/automated	None	Various clinical isolates	Benchmark for MBEC	MBEC	^38–40^
Biofilm Ring Test (BRT)	Static/automated	None	*P. aeruginosa*, *E. coli*, staphylococci	Early biofilm dynamics	MBIC, optionally MBEC	^41–44^
Colony biofilm assay	Static/manual	None	*P. aeruginosa*, *E. coli*	Limited direct PK/PD	cfu counts, biomass quant.	^14,19,55^
Air–liquid interface (ALI)	Static/cell-based	None/human cells	*P. aeruginosa*	Applied in PK/PD trials	cfu, cytokine levels	^56–58^
Drip flow reactor	Dynamic/manual	None	*P. aeruginosa*, multispecies	Nutrient-limited PK/PD	MBEC, biomass, viability	^63,64^
CDC biofilm reactor	Dynamic/manual	None	Staphylococci, *P. aeruginosa*	Flow-based testing	cfu, MBEC, MIC	^65–67^
Rotating wall vessel (RWV)	Dynamic	None	Multispecies	Rarely used for PK/PD	Biomass, cfu	^68–70^
Constant depth film fermenter	Dynamic	None	Oral microbiota	Oral biofilm dynamics	Biofilm thickness, MIC	^71,72^
Membrane perfusion	Dynamic	None	*S. aureus*, *P. aeruginosa*	Drug exposure	cfu, viability	^73^
Microfluidic platforms	Micro-scale Flow	None	*E. coli*, *S. aureus*, mixed	Gradient and response	AUC/MIC, MBIC, cfu, imaging	^44,53,74–76^
BioFlux system (Fluxion)	Microfluidic	None	*S. aureus*, *P. aeruginosa*	Parallel PK/PD testing	MBIC, dynamic viability	^53,79,80^

cfu, colony-forming units; AUC, area under the concentration–time curve; MIC, minimal inhibitory concentration.

^a^Species previously evaluated in the respective models (non-exhaustive); applicability extends to other clinically relevant pathogens.

Across *in vitro* model classes, the trade-off is typically between throughput/standardization (static microtitre-based assays) and exposure realism (flow-based and microfluidic systems), which determines whether conclusions are primarily PD ranking or quantitative PK/PD inference.^14,53,73^

## Ex vivo *models*

*Ex vivo* models bridge the gap between *in vitro* and *in vivo* systems by cultivating biofilms on explanted tissues or organs derived from animals or humans.^25,83^ These models preserve the structural and biochemical complexity of native tissues while allowing for more controlled experimental conditions than *in vivo* studies.^25^ Because they bypass the use of living animals, *ex vivo* models may offer a more ethical and cost-effective alternative for studying host–microbe interactions, tissue-specific colonization and treatment responses.^25,83^

*Ex vivo* models typically use tissues such as skin, cornea, cardiac valves, respiratory epithelium, bones, vaginal mucosa and gastrointestinal tissues as substrates for biofilm growth.^25,84,85^ These substrates closely replicate the *in vivo* microenvironment, including architecture, cellular composition and, in some cases, the presence of host factors like keratin or extracellular matrix proteins.^25,83,86–88^

The following examples therefore represent a selective overview of current approaches, rather than a comprehensive or standardized catalogue.

### Human tissue-derived models

Human skin explants, derived from surgical residues, preserve the structural integrity of the epidermis and dermis.^89^ These models are ideal for studying wound biofilm formation and topical antimicrobial efficacy.^89^ They enable PK/PD analyses within a native tissue environment, enhancing the biological relevance of antimicrobial testing and offering a more realistic assessment of biofilm-associated drug resistance.^89^

### Animal tissue-derived models

The *ex vivo* porcine keratitis model serves as a robust platform for investigating corneal infections and testing antimicrobial therapies. Okurowska *et al.*^90^ introduced a standardized protocol using enucleated pig eyes to model *P. aeruginosa* keratitis, enabling controlled infection and quantitative assessment of treatments. Building upon this, Okurowska *et al.*^91^ demonstrated that *P. aeruginosa* biofilms exhibited significant antibiotic tolerance in this model, underscoring challenges in treating biofilm-associated infections and the importance of PK/PD-informed dosing strategies.

The *ex vivo* oral biofilm model using extracted teeth offers a clinically relevant and reproducible framework for studying multispecies dental biofilms under laboratory conditions.^92^ Guggenheim *et al.*^92^ first described a reproducible system for cultivating multispecies biofilms on human tooth surfaces. Exterkate *et al.*^93^ introduced the Amsterdam Active Attachment (AAA) model, enhancing physiological relevance through continuous nutrient flow. Most recently, Mao *et al.*^94^ employed the AAA model to demonstrate that daily exposure to chlorhexidine and cetylpyridinium chloride significantly modifies microbial community structure and contributes to resistance development.

The *ex vivo* pig lung (EVPL) model has become a key experimental system for simulating cystic fibrosis (CF)-associated pulmonary biofilm infections.^95^ First introduced by Harrison *et al.*^95^, this model utilizes porcine bronchiolar tissue cultured in artificial CF sputum to replicate chronic *P. aeruginosa* infections. Harrington *et al.*^96^ provided a standardized protocol detailing biofilm cultivation and antibiotic testing. More recently, Harrington *et al.*^97^ incorporated transcriptomic profiling, revealing gene expression patterns akin to chronic human infections and highlighting the model's clinical relevance for PD analysis.

*Ex vivo* models have also been used to explore biofilm-associated implant infections.^98,99^ For example, stainless steel tympanostomy tubes, titanium and zirconium implants or osteoblast–biofilm co-culture systems have enabled research into adhesion mechanisms, immune interactions and treatment windows.^98,99^

These models are valuable for pharmacodynamic (PD) and therapeutic window assessments, particularly for investigating time-dependent effects of antibiotics or antibiofilm agents.^100^ In several studies, early-stage biofilms (e.g. within 24 h of formation) showed greater susceptibility to treatment than more mature biofilms, validating current clinical strategies emphasizing early intervention.^85,100^

The respiratory tract is another common focus of *ex vivo* modelling. For instance, *Mycoplasma pulmonis* and *P. aeruginosa* biofilms have been cultivated on tracheal and bronchial explants to replicate the structure and resistance observed in chronic pulmonary infections such as those seen in cystic fibrosis.^101,102^

The genital and gastrointestinal mucosa also serve as substrates for *ex vivo* modelling. The porcine vaginal mucosa closely mimics the human counterpart and has been used to study interactions among *Gardnerella vaginalis*, *Neisseria gonorrhoeae* and *Lactobacillus* spp., as well as biofilm formation by *C. albicans* and *S. aureus.*^103–105^

Similarly, rabbit colonic and ileal tissues have been used to examine biofilms formed by *Aeromonas caviae* and enteroaggregative *E. coli*, helping researchers evaluate tropism, virulence and tissue-specific colonization.^106,107^

Although powerful, *ex vivo* models have important limitations. The absence of an active immune response, competing microbiota and dynamic flow represent key differences compared with *in vivo* infection.^83^ In addition, tissue sterilization and handling can affect tissue viability and microbial growth.^108^ Nevertheless, these constraints are balanced by the ability of *ex vivo* systems to reduce animal use while preserving key structural and matrix-related features of native infection sites.^34,83,108^

### Overview

*Ex vivo* biofilm models preserve relevant structural and matrix characteristics of native tissues and therefore provide a distinct experimental context compared with both *in vitro* assays and *in vivo* infection models. Table 2 summarizes the principal *ex vivo* systems discussed, highlighting their tissue origin, pathogens studied and pharmacological endpoints assessed.

**Table 2.** Overview of *ex vivo* and tissue-based biofilm models used in PK/PD investigations

Model	Category	Host species	Pathogen(s)^a^	Pharmacologic relevance	PK/PD parameters	References
Human skin explant	Skin tissue	Human	*S. aureus*, *P. aeruginosa*	Native matrix response	MBEC, time–kill	^89^
Porcine keratitis model	Ocular tissue	Pig	*P. aeruginosa*	Antibiotic tolerance	MIC, MBEC, cfu	^90,91^
Oral biofilm (tooth, AAA)	Tooth/surface	Human (teeth)	Multispecies oral flora	Antiseptic PK/PD	Biomass, MIC, resistance	^92–94^
EVPL (pig lung, CF model)	Lung tissue	Pig	*P. aeruginosa*	Gene expression + PKPD	AUC/MIC, cfu	^95–97^
Implant infections	Implant/ear	Rat/guinea pig	*S. aureus*, *P. aeruginosa*	PK/PD limited	cfu, adhesion	^98,99^
Respiratory tract infections	Tracheal or bronchial explants	Mice/pig	*M. pulmonis*, *P. aeruginosa*	CF-like biofilm environment; drug penetration, efficacy testing	Qualitative MBEC, potential AUC/MIC assessment	^101,102^
Vaginal mucosa model	Mucosal surface	Pig	*G. vaginalis*, *C. albicans*	Limited PK/PD usage	cfu, histology	^103–105^
GI mucosa (colonic, Ileal)	Mucosal surface	Rabbit	*EAEC*, *A. caviae*	Pathogenic tropism	cfu, adhesion	^106,107^

cfu, colony-forming units; MBIC, minimal biofilm inhibitory concentration; MBEC, minimal biofilm eradication concentration; AUC, area under the concentration–time curve; MIC, minimal inhibitory concentration; CF, cystic fibrosis; EAEC, enteroaggregative *E. coli*.

^a^Species previously evaluated in the respective models (non-exhaustive); applicability extends to other clinically relevant pathogens.

### PK/PD relevance of *ex vivo* biofilm models

*Ex vivo* biofilm models represent an intermediate experimental system between standardized *in vitro* assays and fully integrated *in vivo* infection models, preserving native tissue architecture and biofilm matrix components while allowing controlled experimental manipulation. These models are particularly informative for pharmacodynamic analyses, as they enable assessment of antibiotic time–effect relationships and biofilm tolerance under physiologically relevant diffusion barriers and microenvironmental conditions. However, *ex vivo* systems do not capture systemic pharmacokinetics, and antibiotic exposure at the site of infection is typically defined by externally applied concentrations rather than measured local drug levels. Consequently, while *ex vivo* models are well suited for evaluating pharmacodynamic responses in tissue-associated biofilms, full PK/PD integration requires complementary *in vivo* pharmacokinetic data.^17,18,25,95,97^

## In vivo *models*

*In vivo* biofilm models are essential for analysing host–pathogen interactions, systemic immune responses and the pharmacodynamic behaviour of antimicrobial agents within living organisms. Although numerous models exist to replicate site-specific biofilm infections, standardized protocols are still lacking, complicating comparative assessments and regulatory acceptance.^34^ Recent reviews have highlighted the need for harmonized protocols to improve comparability across studies and facilitate translation into clinical recommendations.^14,109^ Guzmán-Soto *et al.*^14^ critique existing *in vitro* and *in vivo* models, noting that no universal model exists. The review advocates for interdisciplinary approaches to improve model selection for studying biofilms, ultimately aiming to enhance the development of effective antibiofilm therapies.

As with *ex vivo* systems, *in vivo* biofilm models remain highly heterogeneous due to differences in host species, infection sites and pathogens used. This lack of standardization continues to complicate cross-study comparisons and the development of unified evaluation frameworks.

### Human-derived models

#### Oral biofilm (*in situ* plaque models)

The human *in vivo* oral biofilm model, commonly referred to as the *in situ* plaque model, allows investigation of dental plaque formation under natural oral conditions in healthy volunteers. The model was first introduced by von der Fehr *et al.*^110^ in 1970, who used acrylic splints containing enamel slabs worn intraorally to investigate caries development through naturally accumulating plaque.

Subsequent advancements in this methodology have led to the development of standardized protocols. For instance, the Leeds *in situ* device, introduced in the early 2000s, utilized enamel slices mounted on intraoral appliances to study enamel remineralization and biofilm composition over extended periods. This model has been instrumental in evaluating the efficacy of fluoride-containing dentifrices and other therapeutic agents.^111^

More recent studies have continued to refine these models. For example, research conducted in 2022 employed *in situ* devices to assess bacterial colonization on various restorative materials, highlighting significant interindividual variations in microbial composition.^112^ These results highlight the clinical utility of *in situ* models for studying plaque biofilm development and for evaluating restorative and preventive dental materials.

### Animal models by target site or device

#### Lung (CF models in rats/mice)

*In vivo* lung biofilm models in cystic fibrosis (CF) research have advanced to more accurately reflect human pathophysiology, particularly regarding PK/PD parameters of pulmonary antimicrobial therapy. Early models, such as the agar bead model introduced by Cash *et al.*^113^ in 1979, laid the groundwork for studying chronic *P. aeruginosa* infections in rodent lungs.^114^ More recently, the Scnn1b-transgenic (Scnn1b-Tg) mouse model has been developed to replicate CF-like lung pathology, including mucus accumulation and neutrophil infiltration. In this model, *P. aeruginosa* embedded in agar beads is instilled into the lungs, leading to persistent infections that closely resemble human CF lung disease.^115^

Bead- or agar-embedded infection models offer the advantage of reproducibly establishing persistent bacterial infection by delaying clearance and preventing acute sepsis. However, these models also have important limitations: beads represent an artificial foreign material that can induce inflammation independently of bacterial infection, and substantial variability in bead size, number and bacterial load can lead to heterogeneous infection patterns. In addition, the bead matrix does not fully reproduce the complex biological and physicochemical environment of the native airway.^113,116,117^

The Scnn1b-transgenic (β-ENaC) mouse model was first applied for *in vivo P. aeruginosa* lung infections in a study by Brao *et al.*^118^. This study marked the first controlled application of live Scnn1b-Tg mice to investigate pulmonary biofilm infection and antimicrobial response under CF-like conditions.

The C57BL/6 mouse pneumonia model is widely used to investigate lung infections. Borgogna *et al.*^119^ established a standardized intratracheal instillation protocol. Duncan *et al.*^120^ enhanced model fidelity by pre-adapting *P. aeruginosa* in SCFM2, resulting in lung infections that closely reproduce chronic CF lung conditions and enhance translational relevance for PK/PD optimization.^119^

#### Heart (endocarditis models in rats/rabbits)

The rabbit catheter-induced endocarditis model was first described by Garrison and Freedman^121^, who created reproducible cardiac valve vegetations using polyethylene catheter placement and *S. aureus* inoculation. A detailed protocol, often referred to as the ‘Freedman model’, guides catheter placement, bacterial challenge and antibiotic administration to support robust PK/PD analysis of drug penetration into vegetations.^122^ For example, Oramas-Shirey *et al.*^123^ evaluated linezolid versus vancomycin treatment in this model, correlating bacterial clearance with achievable plasma concentrations and MIC values. More recently, Chambers *et al.*^124^ used this model to evaluate daptomycin–β-lactam combinations against daptomycin-nonsusceptible MRSA, demonstrating that optimized combination therapy achieved significantly higher sterilization rates within vegetations, a finding directly tied to improved AUC/MIC pharmacodynamic targets.

#### Ear (chinchilla otitis media)

The chinchilla otitis media model, first established by Giebink^125^, involves intrabulbar or intranasal inoculation with pathogens (e.g. *Streptococcus pneumoniae*), leading to middle ear biofilm formation and inflammation.^126^ Cheung *et al.*^127^ presented a robust SOP for this model, employing microdialysis to monitor real-time antibiotic levels in middle ear fluid (MEF) and plasma, enabling precise PK/PD analyses. Recent work by Yang *et al.*^128^ demonstrated that transtympanic administration of ciprofloxacin via a thermosensitive hydrogel achieved MEF drug levels hundreds of times above the therapeutic level, leading to rapid eradication of pneumococcal biofilms with minimal systemic drug exposure.

#### Eye (keratitis in rabbits/mice)

A few *in vivo* animal models have been developed to study ocular biofilm infections, although integration of PK/PD analyses remains limited. Murine keratitis models employing various mouse strains have demonstrated biofilm formation by *S. aureus*, *P. aeruginosa* and *Fusarium falciforme* on the corneal surface; however, antibiotic penetration and PK/PD analyses have not yet been conducted in these systems.^129–133^ In contrast, rabbit models of *P. aeruginosa* keratitis generated via contaminated soft or rigid contact lenses produce corneal biofilms and significant neutrophilic inflammation; despite this, studies have yet to quantify intraocular antibiotic levels or therapeutic PK/PD indices.^134–137^ These models offer anatomical and pathological relevance to human disease but require future incorporation of robust PK/PD measurements to guide optimized dosing strategies against ocular biofilms.

#### Chronic wounds (wound model in rodents)

*In vivo* chronic wound biofilm models in diabetic and immunocompetent animals provide valuable platforms for PK/PD evaluation of antibiofilm therapies. Zhao *et al.*^138^ first described a diabetic (db/db) mouse model, in which *P. aeruginosa* biofilms delayed wound closure, enabling quantitative analysis of persistent bacterial burden and healing dynamics. Trøstrup *et al.*^139^ refined this approach in C3H/HeN and BALB/c mice using alginate-embedded *P. aeruginosa*, demonstrating heightened inflammation and sustained biofilm presence, a suitable framework for assessing systemic antibiotic levels and AUC/MIC-driven efficacy. Watters *et al.*^140^ further enhanced the model by evaluating antibiotic tolerance in diabetic wounds, noting that biofilm presence markedly reduced drug responsiveness, underscoring the necessity to correlate wound antibiotic levels with bacterial eradication as well as the capacity of insulin to modulate the efficacy of antimicrobial agents. Collectively, these models facilitate PK/PD-informed dosing strategies for chronic wound biofilm management.

#### Bladder/urinary tract (catheter models in rodents)

*In vivo* urinary tract infection models in mice are instrumental for exploring biofilm-related antibiotic efficacy within a PK/PD framework. The classic murine ascending UTI model was first described by Hung *et al.*^141^, enabling precise quantification of bacterial burden in the bladder, kidneys and urine following transurethral *E. coli* inoculation. Later, Brumbaugh *et al.*^142^ demonstrated that maintaining an AUC/MIC ratio ≥100 for novel antimicrobials resulted in efficient eradication of UPEC biofilms in the bladder, underscoring the model’s value in defining clinically relevant PK/PD targets. Reniguntla *et al.*^143^ detailed a refined protocol applying ciprofloxacin treatment schedules and serial bladder/renal sampling to elucidate dose-response relationships. Additionally, nitrofurantoin PK/PD was evaluated in diabetic mouse UTI models, revealing that urine AUC/MIC predicted bacterial load reduction better than time above MIC.^144^

#### Implant-associated (orthopaedic implants in rabbits)

*In vivo* implant-associated biofilm models in animals provide essential platforms for evaluating antimicrobial therapies within a PK/PD framework. A well-characterized example is the rabbit spinal implant infection system described by Gordon *et al.*^145^, in which bilateral pedicle screws and a connecting plate are colonized with bioluminescent *S. aureus*, enabling longitudinal antibiotic efficacy testing through real-time biofilm quantification and imaging. Building on this, Visperas *et al.*^146^ established a standardized rabbit periprosthetic joint infection (PJI) model, detailing surgical implant placement, biofilm inoculation and scheduled debridement and sampling to facilitate robust PK/PD analyses. Complementing these larger animal models, Sokhi *et al.*^147^ developed a mouse tibial implant model using titanium pins and *S. aureus* inoculation to generate persistent biofilms on implants and peri-implant bone. A refined protocol for metal-pin insertion and infection in C57BL/6 mice now allows reproducible infection kinetics and serial sampling.^148^ More recently, Irwin *et al.*^149^ used a rat prosthetic joint model with biofilm-coated pins to quantify *in vivo* bacterial burden and correlate antibiotic exposure with biofilm clearance, underscoring these models’ translational value for optimizing dosing strategies against implant-associated infections.

#### Subcutaneous catheters (rodents)

Subcutaneous catheter-associated biofilm models in rodents provide powerful platforms for evaluating antimicrobial PK/PD against device-associated infections. Kadurugamuwa *et al.*^150^ first reported using bioluminescent *S. aureus* colonized Teflon catheters implanted subcutaneously in mice, enabling real-time, non-invasive monitoring of biofilm development over several weeks (20 days). Allan *et al.*^151^ developed a subcutaneous rabbit model in which chlorhexidine-coated peripherally inserted central catheters (PICCs) significantly reduced *S. aureus* colonization and migration compared to uncoated catheters after thirty days of implantation. More recently, Zhou *et al.*^152^ applied a cefquinome catheter model in mice to link pharmacodynamic indices, such as T>MBIC and AUC/MBIC, with biofilm suppression, revealing biofilm-specific PK/PD targets for treatment optimization. These models facilitate exposure-driven dosing strategies to prevent or eradicate catheter-associated biofilm infections.

*In vivo* biofilm models remain indispensable for elucidating host–pathogen interactions, immune responses and pharmacodynamic behaviour under physiologically relevant conditions. Despite major advances across infection sites, from pulmonary and cardiac to urinary, wound and implant-associated systems, methodological heterogeneity and a lack of standardized protocols continue to hinder comparability and translational alignment. Nevertheless, *in vivo* models uniquely allow the integration of PK/PD analyses within a functioning host environment, making them the benchmark for validating antimicrobial efficacy and optimizing dosing strategies. Their refinement and harmonization across laboratories will be crucial to bridge the gap between experimental findings and clinical application.^14,25,34^

### Overview

Animal models reproduce the complex physiological environment of biofilm-associated infections, enabling assessment of antimicrobial exposure, host immune response and therapeutic efficacy under clinically relevant conditions, as outlined in Table 3. Depending on the infection site, these models are used to study systemic or localized pharmacokinetics, drug penetration and biofilm eradication dynamics.

**Table 3.** Overview of *in vivo* biofilm infection models applied in PK/PD research

Model	Category	Host species	Pathogen (s)^a^	PK/PD relevance	PK/PD parameters	References
*In situ* oral biofilm	Teeth model	Human	Oral flora	Descriptive only	cfu, plaque index	^110–112^
CF lung	Lung model	Mouse/pig	*P. aeruginosa*	PK/PD in mucus	AUC/MIC, cfu, MIC	^113–115,118–120^
Rabbit endocarditis	Heart valve vegetations	Rabbit	*S. aureus*, MRSA	Drug penetration	AUC/MIC, MIC, sterilization	^121–124^
Chinchilla otitis media	Ear infection	Chinchilla	*S. pneumoniae*	Microdialysis possible	AUC/MIC, MEF levels	^125–128^
Keratitis model	Corneal infection	Rabbit/mouse	*P. aeruginosa*, *S. aureus*, *Fusarium*	No PK/PD yet	cfu, histopathology	^129–137^
Diabetic wound (db/db)	Skin/chronic wound	Mouse	*P. aeruginosa*	Antibiotic tolerance	cfu, wound burden	^138–140^
Catheter UTI model	Bladder/kidney	Mouse	*E. coli*, *UPEC*	Defined PK/PD targets	AUC/MIC, T>MIC, MBIC	^141–144^
Implant (rabbit spine/tibial)	Orthopaedic implant	Rabbit/mouse/rat	*S. aureus*	Imaging, MBEC	MBEC, AUC/MIC, cfu	^145–149^
Subcutaneous catheter (rodent)	Device-associated	Mouse/rabbit	*S. aureus*	Real-time tracking	AUC/MBIC, T>MBIC, imaging	^150–152^

cfu, colony-forming units; AUC, area under the concentration–time curve; MIC, minimal inhibitory concentration; MBIC, minimal biofilm inhibitory concentration; MBEC, minimal biofilm eradication concentration; MEF, middle ear fluid; UPEC, uropathogenic *E. coli*; T>MIC, time above MIC.

^a^Species previously evaluated in the respective models (non-exhaustive); applicability extends to other clinically relevant pathogens.

### PK/PD relevance of *in vivo* biofilm models

*In vivo* infection models allow pharmacokinetic exposure and pharmacodynamic response to be evaluated within a functioning host and can support identification of exposure–effect relationships by varying dose and dosing interval to identify PK/PD indices linked to efficacy (e.g. AUC/MIC, C_max_/MIC or time above MIC). However, interpretation depends on whether concentrations at the relevant site of infection are adequately represented, since plasma exposure may not reflect interstitial or tissue concentrations and impaired penetration can contribute to treatment failure. In models such as experimental rabbit endocarditis, antibacterial regimens can be related to clinically relevant exposure (including AUC considerations) and linked to quantitative bacterial burden reductions in infected tissues (including vegetations), thereby supporting translational PK/PD assessment.^23,25,27,124,153^

## Comparison of mentioned models

The following tables (Tables 4–6) summarize the biofilm models presented in this review, organized by *in vitro*, *ex vivo* and *in vivo* classification. For each model, the principal strengths and weaknesses are outlined to highlight their respective advantages and methodological constraints in PK/PD-oriented research.

**Table 4.** *In vitro* biofilm model classification

Model	Strengths	Weaknesses
Microtitre plate (MTP) assays	Widely used, low-cost, scalable, easy to handle; supports high-throughput screening; adaptable to different materials	Lacks flow and shear; static conditions don’t mimic *in vivo* gradients; biomass stains (e.g. crystal violet) don’t differentiate live/dead cells
Calgary Biofilm Device (CBD)	Peg-based system enables standardized MBEC testing; high reproducibility; compatible with many species	No flow; limited surface types; lacks host factors
Biofilm Ring Test (BRT)	Early detection of biofilm via magnetic bead mobility; automated and quantitative	Limited to early biofilm stages; cannot assess mature biofilms or structure
Colony biofilm assay	Nutrient gradients similar to *in vivo*; robust structure; easy to culture on agar filters	No flow or shear; cannot assess antimicrobial penetration; endpoint-only
Air–liquid interface (ALI)	Mimics mucosal interface; supports epithelial co-culture; supports layered biofilms	Technically demanding; requires cell culture expertise; not high-throughput
Drip flow reactor (DFR)	Low-shear gradients; simulates surface-associated chronic biofilms (e.g. wounds)	Non-homogeneous exposure; lower control over biofilm thickness
Rotating wall vessel (RWV)	Simulates low-shear 3D growth and spatial organization	Not standardized; difficult sampling; low oxygen transfer in some designs
Constant depth film fermenter (CDFF)	Maintains biofilm thickness via mechanical control; ideal for oral/dental models	Complex to operate; biofilm heterogeneity possible
Microfluidic platforms	High spatiotemporal resolution; real-time imaging; laminar flow; gradient control	Fabrication complexity; limited tissue integration; expensive

**Table 5.** *Ex vivo* biofilm model classification

Model	Strengths	Weaknesses
Human skin explant	Maintains native architecture; ethically sourced; human relevance	Limited viability over time; no active immune response
Porcine corneal model (keratitis)	Closely mimics human corneal structure; robust infection control	Requires fresh animal tissue; low throughput
Oral biofilm on extracted teeth (AAA Model)	Supports multispecies biofilms; allows antiseptic exposure studies	Inter-donor variation; lacks immune response
*Ex vivo* pig lung (EVPL) model	Recapitulates the CF lung environment; supports chronic biofilms	Labour-intensive; limited availability
Tracheal/bronchiolar tissue models	Preserves 3D airway architecture; suitable for imaging	No immune response; needs fresh tissue
Porcine vaginal mucosa	Human-like mucosa; supports polymicrobial biofilms	Low reproducibility; pH and microbiota sensitive
Gastrointestinal tissue (rabbit ileum/colon)	Suitable for adhesion and colonization assays	Difficult to culture over time; anaerobic growth is complex
Implant-tissue models (e.g. bone or valve)	Simulates clinical implant infections; supports imaging	No circulation; removal and sampling difficult

**Table 6.** *In vivo* biofilm model classification

Model	Strengths	Weaknesses
Human *in situ* plaque model	True host environment; real-time colonization	Ethical/logistical limitations; interindividual variation
Mouse/pig CF lung models	Chronic infections; genetic disease modelling (Scnn1b-Tg)	Ethical concerns; low reproducibility across strains
Endocarditis models (rabbit/rat)	Reproducible vegetations; catheter-based	Invasive surgery; high animal use
Chinchilla otitis media	Anatomically relevant; microdialysis possible	Chinchilla maintenance; middle ear access
Mouse/rabbit keratitis models	Mimics ocular infection; supports histology	Limited PK data; topical drug recovery difficult
Diabetic wound model (mouse)	Chronic wound environment; delayed healing	Variability in wound induction; expensive
Murine UTI/bladder catheter models	Easy catheterization; reproducible cfu counts	Difficult urine PK measurement; short time frames
Orthopaedic implant infection (rabbit/mouse/rat)	Supports longitudinal imaging and sampling	Invasive, needs surgery and sterile implants
Subcutaneous catheter models (mouse/rabbit)	Real-time bioluminescence tracking; easy dosing	Less complex than vascular catheters

### PK/PD relevance and translational considerations

Across model classes, three determinants govern how strongly a study can elucidate PK/PD relationships of antibiotics in biofilms.

1. Exposure realism. Static systems primarily evaluate biofilm PD endpoints at fixed concentrations, which supports comparative ranking but only approximates clinical exposure. Flow-based reactors and microfluidics can generate time-varying exposure and, depending on design, spatial gradients that better reflect biofilm microenvironments.^19,25,26^
2. Site-of-action definition and compartmentalization. *Ex vivo* and *in vivo* models incorporate tissue, mucus and device-related compartments that shape local exposure and therefore support more clinically relevant PK/PD questions. However, these systems often reduce experimental control and throughput, limiting the number of exposure conditions that can be tested.^25,27,154^
3. Readout alignment and quantitative interpretability. Biomass-based readouts are scalable but can decouple from viability and regrowth. Viable-count or regrowth-based endpoints align more directly with bactericidal or suppression objectives but depend on recovery methods and limits of quantification, which can affect comparability across studies.^21,38,53^

Taken together, model selection should be matched to the intended PK/PD use-case: screening and PD ranking (high-throughput static assays), mechanistic exposure–response analysis under controlled gradients (dynamic and microfluidic systems) and translation of dosing strategies (*ex vivo* and *in vivo* models that define compartments and enable clinically relevant sampling).
